# Molecular Genetic Assessment Aids in Clarifying Phylogenetic Status of Iranian Kerman Wild Sheep

**DOI:** 10.3390/ani15020238

**Published:** 2025-01-16

**Authors:** Arsen V. Dotsev, Mohammad Hossein Moradi, Tatiana E. Deniskova, Ali Esmailizadeh, Neckruz F. Bakoev, Olga A. Koshkina, Darren K. Griffin, Michael N. Romanov, Natalia A. Zinovieva

**Affiliations:** 1 L. K. Ernst Federal Research Center for Animal Husbandry, Dubrovitsy, Podolsk 142132, Moscow Oblast, Russia; horarka@yandex.ru (T.E.D.); nekruz82@bk.ru (N.F.B.); olechka1808@list.ru (O.A.K.); n_zinovieva@mail.ru (N.A.Z.); 2 Department of Animal Science, Faculty of Agriculture and Natural Resources, Arak University, Arak 38156-8-8349, Iran; moradi.hosein@gmail.com; 3 Department of Animal Science, University of Tehran, P.O. Box 3158711167-4111, Karaj 31587-1-1167, Iran; 4 Basic Department of Genetic Technologies in Livestock Farming, K. I. Skryabin Moscow State Academy of Veterinary Medicine and Biotechnology, Moscow 109472, Russia; 5 Department of Animal Science, Shahid Bahonar University of Kerman, Kerman 76169-1-4111, Iran; aliesmaili@uk.ac.ir; 6 School of Biosciences, University of Kent, Canterbury CT2 7NJ, Kent, UK; d.k.griffin@kent.ac.uk; 7 Animal Genomics and Bioresource Research Unit (AGB Research Unit), Faculty of Science, Kasetsart University, Chatuchak, Bangkok 10900, Thailand

**Keywords:** Kerman wild sheep, Asiatic mouflon (*Ovis gmelini*), urial (*Ovis vignei*), population structure, genetic diversity, SNPs, mitochondrial DNA, phylogenetics

## Abstract

Two species of wild sheep inhabit Iran: Asiatic mouflon (*Ovis gmelini*) and urial (*O. vignei*). Phylogenetic relationships between populations distributed in this country are complex and still remain unclear. This study aimed to clarify, by genetic assessment, the phylogenetic status of Kerman wild sheep, considered to be a hybrid of the two species. For this purpose, we created a dataset that included specimens of *O. gmelini*, *O. vignei*, and Kerman sheep. We applied genome-wide SNP genotyping technology to analyze population structure and genetic diversity of these groups. Using Neighbor-Net and PCA plots, it was demonstrated that Kerman sheep were differentiated from other groups and occupy an intermediate position between *O. gmelini* and *O. vignei*. Using Admixture analysis, two ancestral components were identified in this population; however, admixed ancestry was not confirmed by f3 statistics. Genetic diversity in Kerman wild sheep was significantly higher than in any group of *O. vignei,* but lower than in *O. gmelini.* Additionally, we examined complete mitochondrial genomes and it was demonstrated that the matrilineal ancestor of Kerman sheep belonged to *O. vignei*. Our results lead to the conclusion that Kerman wild sheep can be recognized as a separate subspecies of *O. vignei*.

## Simple Summary

Asiatic mouflon (*Ovis gmelini*) and urial (*O. vignei*) are two species of wild sheep that occur throughout the mountains of Iran. At the moment, phylogenetic (“family tree”) relationships between populations of wild sheep in this region remain unclear. Three subspecies of the Asiatic mouflon and three subspecies of the urial were described in Iran. In our study, utilizing molecular genetic tools, we, for the first time, investigated the phylogenetic status of the Kerman wild sheep, as this has long been considered to be a hybrid of Asiatic mouflon and urial. We examined three specimens of Kerman sheep using nuclear and mitochondrial DNA approaches. Our results demonstrated that Kerman sheep were different from other groups and occupy an intermediate position between the two wild breeds. We demonstrated that the maternal line ancestor of the Kerman sheep belonged to the urial. In our opinion, therefore, Kerman wild sheep can be recognized as a separate subspecies of the urial.

## 1. Introduction

The mountainous regions of Iran are home to a number of populations of wild sheep that belong to two species: Asiatic mouflon (*Ovis gmelini*) and urial (*O. vignei*). Currently, these ungulates are assessed on the IUCN Red List as “Near Threatened” and “Vulnerable”, respectively [1,2]. The census size of these species has been declining over recent decades due to poaching, competition with livestock, and habitat deterioration.

Special interest in *O. gmelini* is also based on the fact that this species is considered to be the ancestor of domestic sheep (*O. aries*). Domestication of this important agricultural animal occurred around 10,000 years BC on the territory of Fertile Crescent, spanning present-day Iran, Turkey, Syria, and neighboring countries [3].

Understanding phylogeny is crucial in the development of strategies for the conservation of animals. Relationships between populations of wild sheep in Iran, however, still remain unclear. For a long period of time, the mouflon and urial were combined into one species, i.e., *O. orientalis*, due to their similarity in morphology and the ability of interbreeding to produce fertile offspring. However, cytogenetic analysis revealed that these groups of animals have different numbers of chromosomes, i.e., 2*n* = 54 in the mouflon and 2*n* = 58 in the urial [4,5]. According to the International Council for Game and Wildlife Conservation (CIC) [6], three subspecies of the mouflon, i.e., Armenian (*O. g. gmelini*), Esfahan (*O. g. isphahanica*), and Laristan (*O. g. laristanica*), and three subspecies of the urial, including Transcaspian (*O. v. arkal*), Afghan (*O. v. cycloceros*), and Blanford’s (*O. v. blanfordi*), inhabit Iran. Mouflon populations are located in the western parts and urials are distributed in the eastern parts of the country. A hybrid form between two subspecies of the mouflon, i.e., Shiraz (*O. g. gmelini* × *O. g. laristanica*) and two interspecific hybrids, including, Alborz red sheep (*O. g. gmelini* × *O. v. arkal*) and Kerman wild sheep (*O. g. laristanica* × *O. v. blanfordi*), were also described.

**Figure 1:**
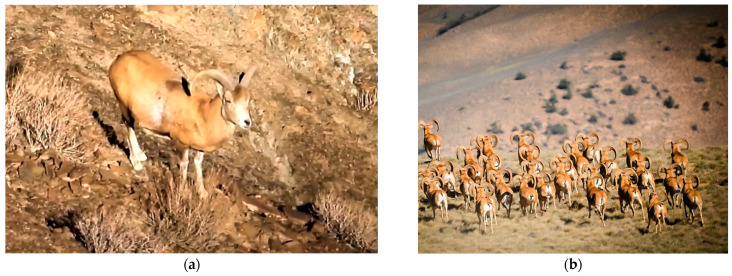
Kerman wild sheep in their natural habitat. (**a**) One of the specimens (WU950/PQ652216) examined in this study. Courtesy: A documentary screenshot by Sergey Mazurkevich. (**b**) A herd of wild sheep in Khabr National Park in Kerman province, Southern Iran. Credit: https://commons.wikimedia.org/wiki/File:Khabr_national_park.jpg, accessed on 4 December 2024, by Sina.najmadini (CC-BY-SA-4.0).

Kerman wild sheep (Figure 1) inhabit the Khabr National Park and Baft Mountains in Kerman and Yazd provinces of Iran. This territory is located between the ranges of *O. gmelini* and *O. vignei*. In terms of size, the Kerman sheep is slightly larger than *O. gmelini*. The coat color is mostly pale brown with a whitish saddle-shaped patch. The horns are homonymous with a flat frontal surface, reaching an average length of around 78 cm in mature rams [6]. Little is known about phylogeny of this population; however, it was shown that the Kerman sheep karyotype consists of 2*n* = 54 and 2*n* = 55 chromosomes [7].

In recent times, an increasing number of long-standing phylogenetic mysteries in the Caprinae subfamily were unraveled by molecular genetic techniques. In particular, recently, based on examination of mitochondrial DNA (mtDNA), a taxonomic reassessment of gorals (*Naemorhedus*) was conducted [8,9]. The taxonomic status of the serow (*Capricornis*) was analyzed at the whole-genomic level [10]. Utilizing genome-wide SNP analysis [11] and exploration of the mitochondrial gene *cytb* [12], a new species of the snow sheep (*O. nivicola*) was proposed. Given that the taxonomic status of the Kerman wild sheep remains unclear, however, the aim of the current study was to assess the phylogenetic relationship of this small ruminant compared to other closely related populations using both nuclear and mtDNA sequences.

## 2. Materials and Methods

### 2.1. Ethics Statement

All the samples examined in this study were harvested legally by trophy hunters in Iran, Pakistan, Tajikistan, and Uzbekistan. During the expeditions, all the regulations of the respective countries were followed. The protocol for the study, No. 2 (28 April 2022), was approved by the Commission on the Ethics of Animal Experiments of the L.K. Ernst Federal Research Center for Animal Husbandry.

### 2.2. Animals, Samples, and DNA Extraction

**Figure 2:**
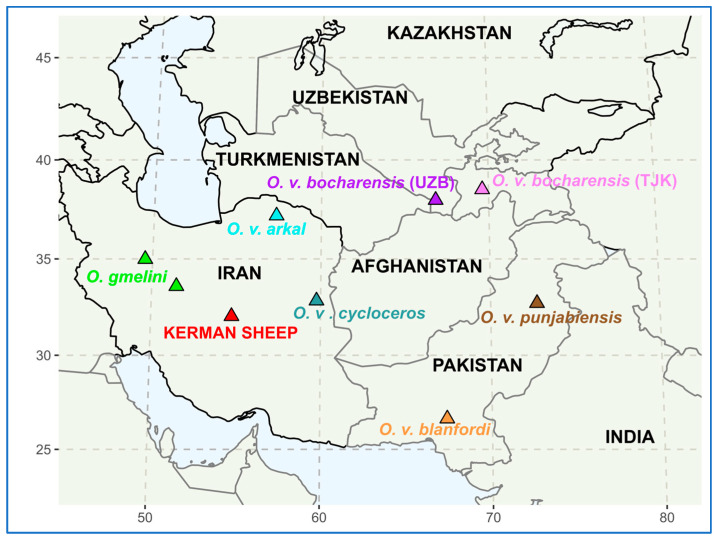
The sampling sites of the specimens examined in this study. Abbreviations: *O.*, *Ovis*; *O. v.*, *Ovis vignei*; UZB, Uzbekistan; TJK, Tajikistan.

Fifteen specimens of the urial belonging to five subspecies, i.e., Transcaspian (*O. v. arkal*, *n* = 2), Afghan (*O. v. cycloceros*, *n* = 2), Blanford’s (*O. v. blanfordi*, *n* = 3), Punjab (*O. v. punjabiensis*, *n* = 3), and Bukhara (*O. v. bocharensis*, *n* = 5), and five specimens of the Asiatic mouflon (*O. gmelini*) from Iran were examined. The Bukhara urial was regarded as two different populations, one from Tajikistan (*n* = 3) and the other from Uzbekistan (*n* = 2). The samples of the Kerman wild sheep were represented by three specimens from Yazd province, Iran. The list of the samples used in this research is given in Table 1 and the geographic distribution map of the sampling sites is shown in Figure 2. The latter was created using R packages maps [13] and ggplot2 (version 3.3.2) [14].

DNA isolation was undertaken from muscle tissues using Nexttec columns (Nexttec Biotechnology GmbH, Leverkusen, Germany).

### 2.3. Genome-Wide SNP Genotyping

To study the phylogenic relationship between Kerman wild sheep and other closely related populations based on nuclear DNA analysis, we employed genome-wide SNP genotyping using the following two Illumina (San Diego, CA, USA) Ovine BeadChips: OvineSNP50 BeadChip and Ovine Infinium^®^ HD SNP BeadChip 600K, containing around 50,000 and 600,000 SNPs, respectively.

The generated datasets from the two DNA chips were merged and only common loci were selected for the subsequent analyses. After the primary quality control procedures, SNPs with GT and GC scores lower than 0.5 were excluded. The subsequent filtering steps were performed in PLINK 1.9 software [20] when SNPs were genotyped in less than 90% (--geno 0.1) of the samples, with minor allele frequencies less than 5% (--maf 0.05), and showing linkage disequilibrium (--indep-pairwise 50 5 0.5) were removed. All the samples included in the study were checked for the level of successfully genotyped SNPs and passed a threshold of 90% (--mind 0.1). PLINK 1.9 was also used for calculations of principal component analysis (PCA) procedure (--pca) and identical-by-state (IBS) genetic distances (--distance 1-ibs square). Pairwise *F*_ST_ genetic distances were calculated in the R package StAMMP [21]. The construction of Neighbor-Net graphs based on IBD and pairwise *F*_ST_ genetic distances was performed in SplitsTree 4.14.6 software [22]. Cluster analysis was carried out using program Admixture 1.3 [23]. To test whether a target population has an admixed ancestry, we applied f3 statistics in the R package admixr [24]. Genetic diversity was assessed using the R package inbreedR [25] by calculating multilocus heterozygosity (MLH), defined as a ratio of the number of heterozygous loci in an individual to a total number of loci that were selected for the study. The visualization of the PCA and MLH plots was performed with the use of the R package ggplot2 (version 3.3.2) [14].

### 2.4. Complete Mitochondrial Genome Sequencing

To infer phylogeny based on maternal inheritance, we examined complete mitochondrial genomes of seven urials, six Asiatic mouflons from Iran, and five domestic sheep representing all the described haplogroups. Two samples of the argali (*O. ammon*) were taken as an outgroup (Table 1).

Complete mitochondrial genomes of the Kerman wild sheep and Bukhara urial were derived and assembled from the whole genome sequences using BWA-MEM2 [26] and bcftools 1.19 [27]. The whole genome sequencing data with a 20× coverage (55 Gb) was obtained using an Illumina HiSeq platform. The generated number of mitochondrial DNA reads allowed us to assemble complete genomes with a read depth (DP) more than 200×. The other mitogenomes of *O. vignei*, *O. gmelini*, *O. aries*, and *O. ammon* that we used in this research were retrieved from GenBank and their accession numbers are given in Table 1.

For phylogenetic analysis, we used concatenated sequences of 2 rRNA and 13 protein-coding genes that were annotated with Mitos2 [28]. The best-fit models of evolution for nucleotides were determined in PartitionFinder2 software [29]. The Bayesian phylogenetic tree was constructed using the MrBayes 3.2.6 program [30]. For the visualization of the phylogenetic tree, Figtree 1.4.2 software [31] was applied.

## 3. Results and Discussion

In this study, we, for the first time, conducted a molecular genetic assessment of the phylogenetic status of Iranian Kerman wild sheep. Despite the fact that we had small sample sizes in our groups, objective results were nonetheless generated for both nuclear and mtDNA. The studies on nuclear DNA make it possible to trace more recent evolutionary events, since an individual receives their genetic information from both parents. This approach can help detect events of hybridization and Admixture between populations. Here, we applied genome-wide SNP genotyping using Illumina Ovine BeadChips that were developed for research on domestic sheep. Previously, this approach was efficiently applied for the following different wild and feral species of the genus Ovis: bighorn (*O. canadensis*) [32], thinhorn (*O. dalli*) [33], snow sheep (*O. nivicola*) [11], argali (*O. ammon*) [15], Asian mouflon [34], and European mouflon (*O. aries musimon*) [35].

In contrast, mtDNA is transmitted only along the maternal lineage and does not undergo recombination. Therefore, it allows us to obtain information on more distant ancestry. In the present study, using mtDNA, we determined which species the matrilineal ancestor to which the Kerman sheep could belong.

### 3.1. Genome-Wide SNP Genotyping and Patterns of Relationships

**Figure 3:**
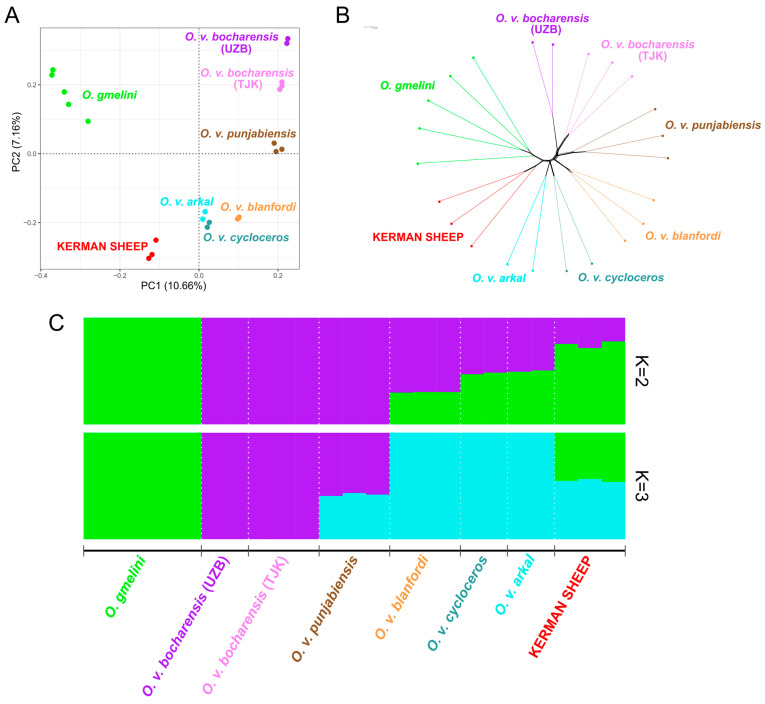
Principal component analysis-based plot (**A**), an individual Neighbor-Net tree (**B**), and Admixture analysis-assisted plot (**C**) revealing population structure in *O. gmelini* and *O. vignei*. This figure includes the Asiatic mouflon (*O. gmelini*), three subspecies of the urial, i.e., Transcaspian (*O. v. arkal*), Afghan (*O. v. cycloceros*), and Blanford’s (*O. v. blanfordi*), from Iran as well as three other subspecies of the urial, including Punjab (*O. v. punjabiensis*, Pakistan) and Bukhara (*O. v. bocharensis*, from Tajikistan and Uzbekistan). Abbreviations: UZB, Uzbekistan; TJK, Tajikistan.

A total of 30,234 SNPs passed quality control filters and were selected for the subsequent investigation. PCA output (Figure 3A) demonstrated that the studied groups formed seven clusters, and all the samples were assigned to their respective populations. Principal component PC1 that explained 10.66% of the genetic variance that divided urials (PC1 > 0) from mouflons (PC1 < 0). The Kerman wild sheep were placed on PC1 together with mouflons and were separated from them by PC2 and PC3 (Supplementary Figure S1), which explained 7.16% and 6.45% of the genetic variance, respectively. The closest populations to the Kerman sheep were urials from Iran, i.e., *O. v. arkal* and *O. v. cycloceros*. An individual phylogenetic tree (Neighbor-Net) followed a similar pattern (Figure 3B). All the studied groups of urials were clustered separately from each other and clearly differentiated from mouflons. The branch containing samples of the Kerman wild sheep was located between the *O. v. arkal* and *O. gmelini* branches.

At K = 2, the cluster analysis performed using the Admixture 1.3 software (Figure 3C) separated *O. gmelini* from *O. v. bocharensis* and *O. v. punjabiensis*, while the other populations consisted of two genetic components. At K = 3, the ancestral component was also revealed for *O. v. blanfordi*, *O. v. cycloceros*, and *O. v. arkal*. The Kerman sheep was identified as an admixed group between *O. gmelini* and *O. vignei*. However, it should be noted that the lowest cross-validation error for our dataset was found at K = 1 (Supplementary Figure S2) and it implies that this analysis could not detect strong genetic differentiation between the studied groups. To identify whether the Kerman sheep has an admixed origin, we also implemented f3 statistics. As a result, we obtained only positive values in all cases when the Kerman sheep was regarded as a source population and, therefore, this analysis did not confirm that hypothesis.

Until now, the Kerman wild sheep was not regarded as a valid subspecies, mostly based on the study of its karyotype where a hybrid origin of this population was proposed. We surmise, however, that it should be mentioned here that the numbers of chromosomes vary significantly in different species of the genus *Ovis*, from 2*n* = 52 in the snow sheep (*O. nivicola*) to 2*n* = 58 in the urial (*O. vignei*), and this does not reflect the phylogenetic relationships between the species. For example, the number of chromosomes in *O. gmelini* (2*n* = 54) is different from phylogenetically close *O. vignei* and identical to phylogenetically distant North American wild sheep, i.e., thinhorn (*O. dalli*) and bighorn (*O. canadensis*). Therefore, we suggest that much more informative methods currently available, such as genome-wide genotyping and sequencing, should be applied in analyzing the taxonomy and evolution of the genus *Ovis*.

**Figure 4:**
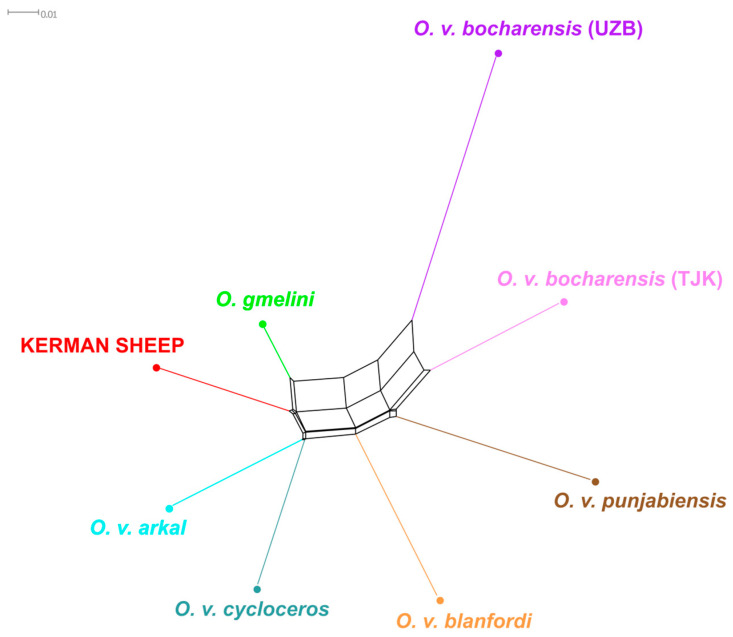
Neighbor-Net configuration demonstrating phylogenetic relationships of *O. gmelini* and *O. vignei* populations based on pairwise *F*_ST_ genetic distances. Abbreviations: UZB, Uzbekistan; TJK, Tajikistan.

The analysis of population differentiation based on pairwise *F*_ST_ genetic distances (Supplementary Table S1) revealed that the estimates between the subspecies of *O. vignei* ranged from 0.099 for the *O. v. arkal* and *O. v. cycloceros* pair to 0.205 that was observed between those subspecies and *O. v. bocharensis* from Uzbekistan. The *F*_ST_ values between *O. gmelini* and *O. vignei* populations varied from 0.089 (*O. v. arkal*) to 0.156 (*O. v. bocharensis* from Uzbekistan). The closest groups to the Kerman sheep were *O. gmelini* (0.076), *O. v. cycloceros* (0.104), and *O. v. arkal* (0.109). The pairwise *F*_ST_ genetic distances were also used for the construction of the Neighbor-Net tree (Figure 4).

As can be observed in Figure 4, the subspecies of *O. vignei* formed branches according to their geographical distribution and the populations from Iran were genetically closest to *O. gmelini*. The most distant population from *O. gmelini* among urials was *O. v. bocharensis*. The Kerman sheep formed a branch that was located between *O. gmelini* and *O. vignei*.

**Figure 5:**
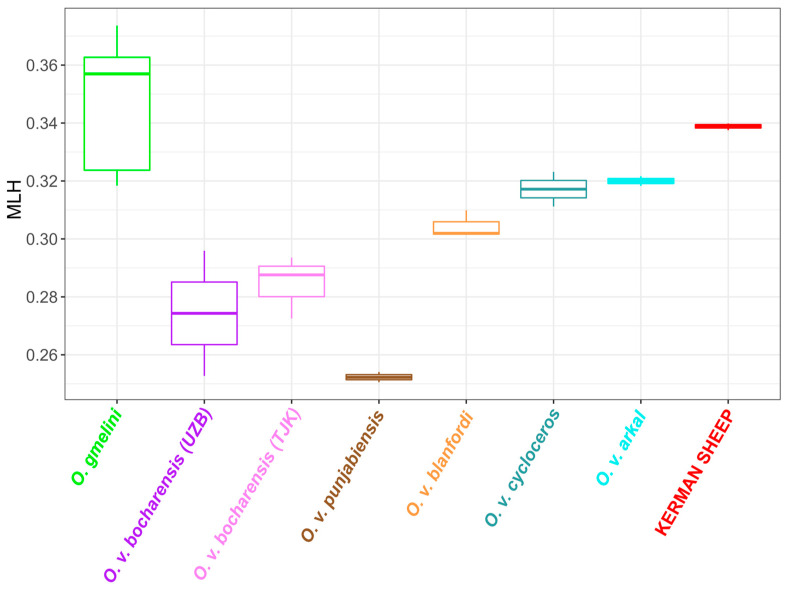
Multilocus heterozygosity (MLH) in Kerman wild sheep as compared to groups of *O. gmelini* and *O. vignei*. Abbreviations: UZB, Uzbekistan; TJK, Tajikistan.

When assessing genetic diversity using MLH calculation (Supplementary Table S2), we observed higher MLH estimates in *O. gmelini* than in *O. vignei* (Figure 5). This, however, should be regarded with caution since it could be affected by ascertainment bias. The Illumina Ovine BeadChips used in this study were developed for domestic sheep (*O. aries*), and *O. gmelini* are genetically closer to this species than to *O. vignei*. Among *O. vignei* subspecies, the highest MLH values were detected for populations from Iran, i.e., *O. v. arkal* and *O. v. cycloceros*, and the lowest one for *O. v. punjabiensis*. Heterozygosity in the Kerman wild sheep was significantly higher than in any group of *O. vignei* but lower than in *O. gmelini*.

### 3.2. Complete Mitochondrial Genomes

**Figure 6:**
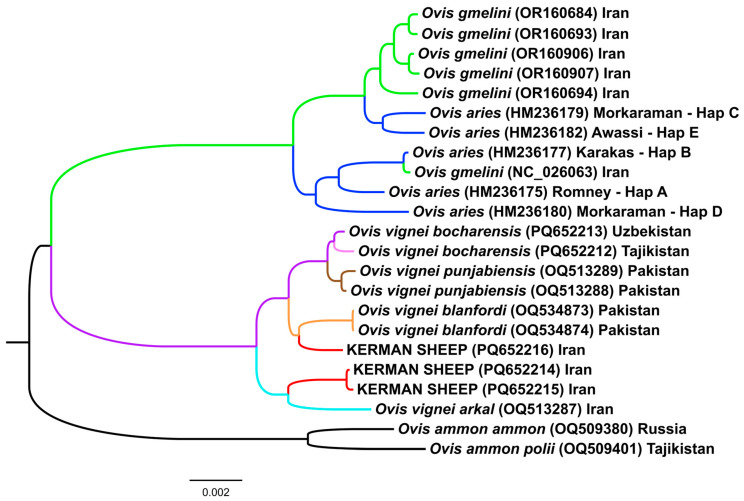
Rooted Bayesian phylogenetic tree based on mitogenomes. All posterior probabilities were equal to 1.

To study phylogeny based on mtDNA, we extracted three mitogenomes of the Kerman wild sheep as well as two mitogenomes of the Bukhara urial from whole genome sequencing data. We added to our dataset samples of mouflons, domestic sheep, urials, and argali from the NCBI GenBank (Table 1). We constructed a Bayesian phylogenetic tree (Figure 6) using concatenated sequences that consisted of two mitochondrial rRNA and 13 protein-coding genes, which were 13,875 bp in length. A very strong posterior probability support of 1 for all the nodes of the phylogenetic tree was detected.

Three major clades were identified as follows: Clade 1 that included samples of *O. ammon* taken as an outgroup, Clade 2 that consisted of samples of *O. vignei*, and Clade 3 that contained samples of *O. gmelini* and *O. aries*. All the specimens of the Kerman wild sheep belonged to Clade 2 formed by *O. vignei*. Two of them were placed in the subclade together with *O. vignei arkal* and the other one was found in the subclade along with representatives of *O. vignei blanfordi*.

### 3.3. Interpretation of Molecular Genetic Data Produced for Kerman Sheep

Our research on genome-wide SNP genotyping demonstrated that *O. gmelini* and *O. vignei* were very close genetically. The highest pairwise *F*_ST_ genetic distances indicated moderate differences between these species as the values varied from 0.089 to 0.156. It should be noted that *F*_ST_ values were higher even among some subspecies of *O. vignei* (up to 0.205). Moreover, the cross-validation error calculated for the Admixture analysis suggested that the number of ancestral populations for our dataset was equal to 1 (K = 1). In contrast, the investigation of complete mitochondrial genomes showed clear differentiation of *O. gmelini* and *O. vignei*.

All the samples of the Kerman sheep were assigned to their own cluster on the PCA plot (Figure 3A) and had their own branch in the Neighbor-Net tree (Figure 3B). In Admixture analysis (Figure 3C), at K = 2 and K = 3, we observed *O. gmelini* and *O. vignei* ancestry in the Kerman sheep. However, f3 statistics did not detect admixed ancestry in this population. Using mtDNA analysis, it was determined that the Kerman sheep maternal line originated from *O. vignei*. Therefore, in our opinion, the Kerman sheep can be recognized as a subspecies of *O. vignei*.

## 4. Conclusions

Molecular genetic studies of the phylogenetic status of the Kerman wild sheep from Iran demonstrated that this population is genetically differentiated from other groups of *O. vignei* and *O. gmelini*. Genome-wide SNP analysis revealed that the Kerman wild sheep occupies an intermediate position between these two species. Using Admixture analysis, we found evidence that this population had an admixed ancestry; however, this was not supported by f3 statistics. The examination of complete mitochondrial genomes indicated that the Kerman wild sheep belongs to *O. vignei*. Overall, our findings lead to the conclusion that the Kerman wild sheep can be recognized as a subspecies of *O. vignei*. We suggest, however, that additional studies based on whole genome sequencing and a larger number of samples are needed for the final recognition that the Kerman sheep can be considered a separate subspecies.

## Figures and Tables

**Table 1:** The list of samples that were examined in this study.

Species/Population	Country	Genome-Wide SNP Genotyping		Complete Mitochondrial Genome Analysis		
		*n* ^1^	Reference	*n*	GenBank Accession Number	Reference
Kerman wild sheep	Iran	3	This study	3	PQ652214, PQ652215, PQ652216	This study
Transcaspian urial (*O. v. arkal*)	Iran	2	[15]	1	OQ513287	[15]
Afghan urial (*O. v. cycloceros*)	Iran	2	[15]	ND ^2^	ND	ND
Blanford’s urial (*O. v. blanfordi*)	Pakistan	3	[15]	2	OQ534873, OQ534874	[15]
Punjab urial (*O. v. punjabiensis*)	Pakistan	3	[15]	2	OQ513288, OQ513289	[15]
Bukhara urial (*O. v. bocharensis*)	Tajikistan	3	This study	1	PQ652212	This study
Bukhara urial (*O. v. bocharensis*)	Uzbekistan	2	This study	1	PQ652213	This study
Asiatic mouflon (*O. gmelini*) (unspecified)	Iran	5	This study (*n* = 3), [16] (*n* = 2)	6	KF938360, OR160684, OR160693, OR160694, OR160906, OR160907	[17,18]
Domestic sheep (*O. aries*)	ND	ND	ND	5	HM236175, HM236177, HM236179, HM236180, HM236182	[19]
Altai argali (*O. ammon ammon*)	Russia	ND	ND	1	OQ509380	[15]
Pamir argali (*O. ammon polii*)	Tajikistan	ND	ND	1	OQ509401	[15]

^1^ *n*, number of samples; ^2^ ND, no data available.

## Data Availability

SNP data in PLINK format were deposited to Figshare: https://doi.org/10.6084/m9.figshare.28124693.v1. The complete mitochondrial genomes of the Kerman wild sheep and Bukhara urials were deposited into NCBI GenBank (https://www.ncbi.nlm.nih.gov/nuccore, accessed on 4 December 2024) under accession numbers PQ652212 to PQ652216.

## Supplementary Materials

The following supporting information can be downloaded at: https://www.mdpi.com/article/10.3390/ani15020238/s1. Figure S1: Principal component analysis (PC1–PC3) revealing population structure in *O. gmelini* and *O. vignei*; Figure S2: Cross-validation error plot for Admixture analysis; Table S1: Pairwise *F*_ST_ genetic distances between populations of *O. gmelini* and *O. vignei*; Table S2: Values of multilocus heterozygosity (MLH) in studied specimens.
