# Supplementary material for: Molecular Genetic Assessment Aids in Clarifying Phylogenetic Status of Iranian Kerman Wild Sheep
Source: Animals (Basel). 2025 Jan 16;15(2):238. doi: 10.3390/ani15020238 (PMC11758319; doi:10.3390/ani15020238)
Supplement: Supplementary file 1 [file animals-15-00238-s001.zip › Supplementary Materials.pdf]

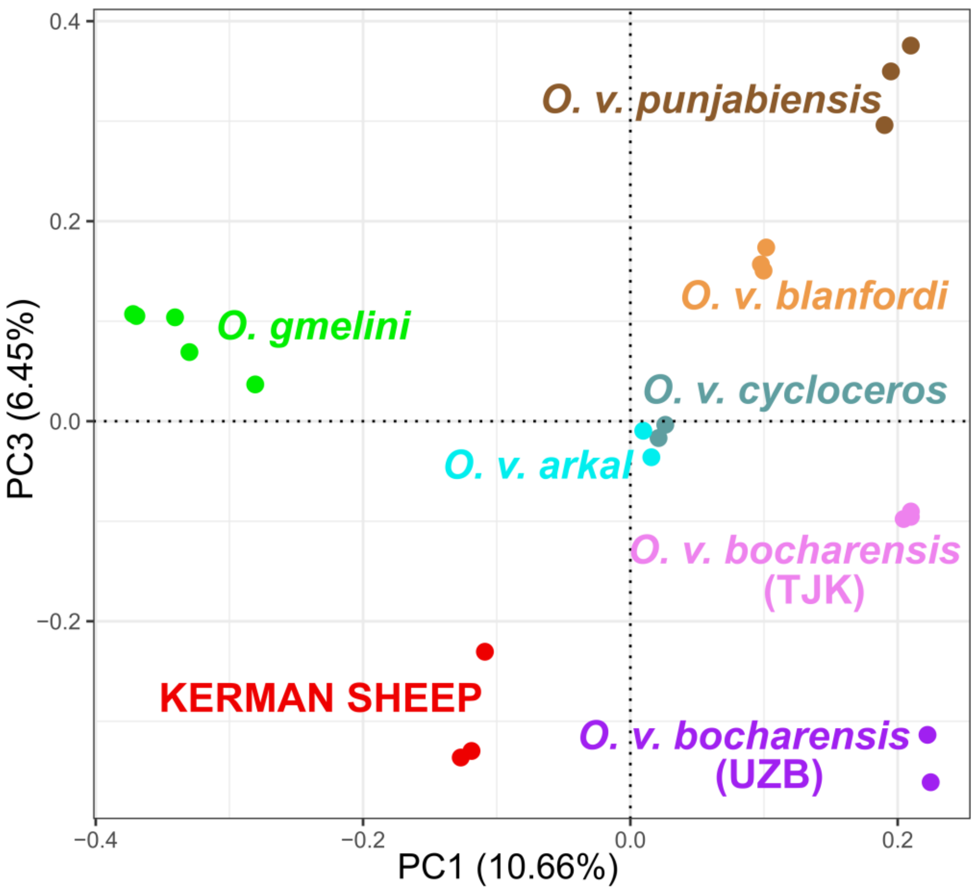

Figure S1. Principal component analysis (PC1–PC3) revealing population structure in *O. gmelini* and *O. vignei*.

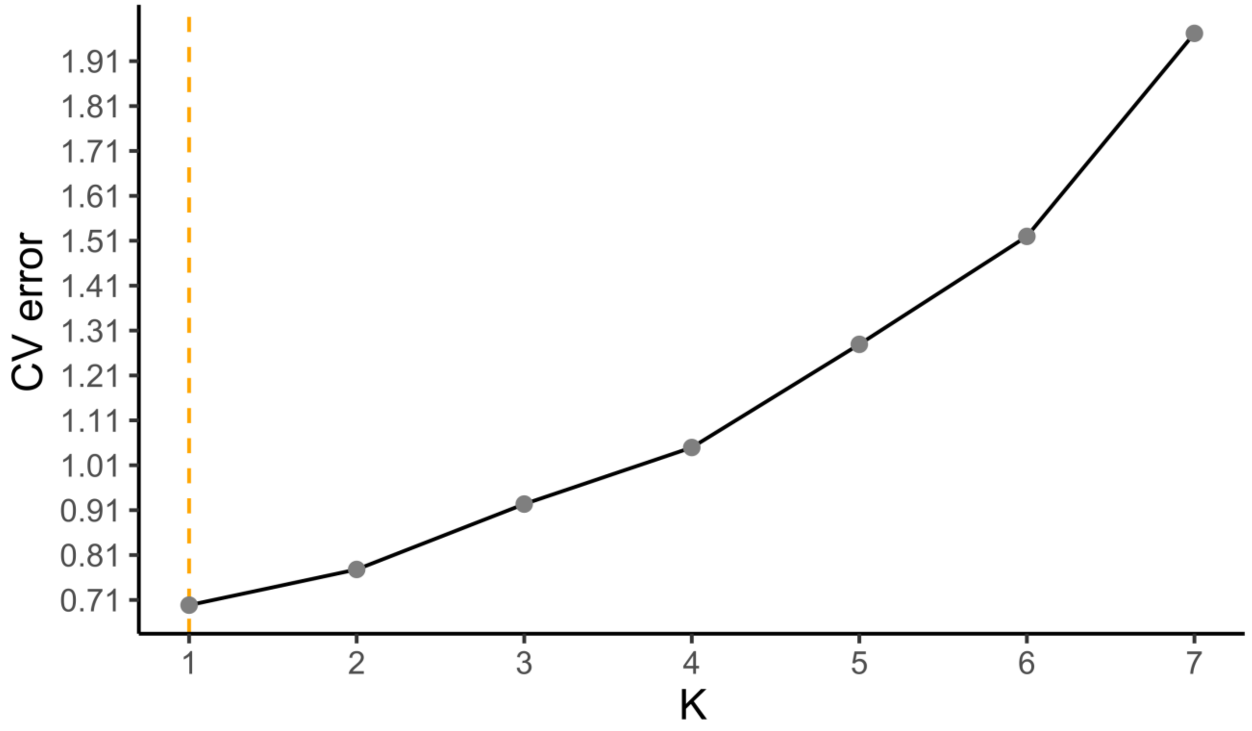

Figure S2. Cross-validation error plot for the Admixture analysis.

Table S1. Pairwise F_{ST} genetic distances between populations of *O. gmelini* and *O. vignei*.

(See the Excel file Supplementary Table S1.xlsx).

Table S2. The values of multilocus heterozygosity (MLH) in the studied specimens.

Group	Sample ID	MLH
O. gmelini	OGM_ESF_WU1092	0.373624513
O. gmelini	OGM_ARM_WU1058	0.356966311
O. gmelini	OGM_ARM_WU1059	0.362688336
O. gmelini	OOR_AMF1	0.323717623
O. gmelini	OOR_AMF4	0.318401896
O. v. bocharensis (UZB)	OVI_BUKH_WU1013	0.295920095
O. v. bocharensis (UZB)	OVI_UZB_WU1251	0.252717115
O. v. bocharensis (TJK)	OVI_BUH_365	0.293583884
O. v. bocharensis (TJK)	OVI_BUH_425	0.272557982
O. v. bocharensis (TJK)	OVI_TJK_WU1255	0.287590994
O. v. punjabiensis	OVI_PUNJ_575	0.252243101
O. v. punjabiensis	OVI_PUNJ_576	0.254071441
O. v. punjabiensis	OVI_PUNJ_960	0.250550195
O. v. blanfordi	OVI_BLN_361	0.301946843
O. v. blanfordi	OVI_BLN_377	0.309903504
O. v. blanfordi	OVI_BLN_961	0.301371254
O. v. cycloceros	OVI_IRAN_WU880	0.323175893
O. v. cycloceros	OVI_IRAN_WU1093	0.311190113
O. v. arkal	OVI_IRAN_WU1094	0.31833418
O. v. arkal	OVI_IRAN_WU1097	0.321618419
KERMAN SHEEP	KERMAN_430	0.337531742
KERMAN SHEEP	KERMAN_728	0.339055358
KERMAN SHEEP	KERMAN_WU950	0.339867953

Note: UZB, Uzbekistan; TJK, Tajikistan.
